# The Uses of a Syllabus

**Published:** 1921-08-06

**Authors:** 


### THE MATRONS' AND SISTERS' DEPARTMENT.

### THE USES OF A SYLLABUS.

Considering recent advances in the art of teaching, it is certainly astonishing that many persons should have been found so totally ignorant of the meaning of a syllabus as would appear from the objections which have been made to that issued by the General Nursing Council of England and Wales. All who are concerned with preparing candidates directly or indirectly for examinations are confronted with the necessity for observing the conditions laid down in their syllabus. It indicates the subjects on which instruction will be given. It is a useful indication of the special qualifications required of the teacher, and it gives the pupil some general notion of the branches of knowledge he will be called upon to acquire. If intelligently drawn up it indicates the order in which the prescribed course of study should be imparted, and it may help the teacher materially by pointing out what subjects are correlated and may best be dealt with in close connection.

It is a marked property of all the syllabuses with which we are acquainted that they terrify the unlearned. They are perforce drawn up in technical language. They deal in generalities. They leave much to the imagination. They convey to the ignorant the impression that every subject mentioned is to be followed up in all its ramifications. Hence the pride and gratification of the "V.A.D." when, after satisfying the local doctor who has gently explored the depths of her ignorance, she can display to admiring friends the syllabus on which her studies have been framed. So little is the art of syllabus-making apprehended that we have seen but recently the preposterous comment made upon the Draft Syllabus that any well-educated girl could pass it (*sic*) without having seen the inside of a hospital.

The absurdity of confusing this well-thought-out scheme of instruction with a standard of knowledge on the subjects it names can be seen by examining it in detail. In the first year we find such subjects as elementary pharmacology, metabolism, reproductive system, communicable and notifiable diseases, gynæcological nursing, etc. In each of these subjects the merest fringe could be touched on in lecturing to first-year probationers, but, none the less, it is of great importance that some impression should be given of the department which the words indicate. The difference in the attitude of the pupil towards such branches of knowledge which one well-planned lecture can make is incalculable. It has been usual for the student to be left to gather unaided what impressions she can of the numerous matters relating to her ward work which she is not fitted fully to grasp during her early hospital experiences. The syllabus aims at giving such a comprehensive and cursory view of her field of action as will enable her to fit in point by point all that she learns by experience and observation on her way through the hospital.

Nothing but long observation of the ways of probationers when confronted with disease in all its numerous ramifications for the first time could have evolved the wise *résumé* of preparatory study which is to be found in the Draft Syllabus. In effect it is an attempt to go before the puzzled girl who is trying to become a nurse and remove some of the worst obstacles which lie in her path. At every turn she needs a chart of the journey before her. It is not so much that she needs at this stage to know anything thoroughly, but she does need to be shown what she has to learn. She needs to be protected from forming wrong notions. She needs a foundation on which she can erect a solid edifice of sound information. She must, in short, be aided to grasp the whole before with awakened perceptions she can set herself profitably to study the parts. It is this which marks the value of the first year's work so ably summarised in the Draft Syllabus, and those who scorn it have failed to appreciate the amazing reformation it is capable of effecting in the training of nurses. It was long ago found out that the lectures delivered by doctors to nurses during their first year are wasted in nine cases out of ten. In the hands of an able sister or matron it will be discovered after one or two trials what admirable scope the Draft Syllabus gives for individual teaching, for diversity of method and for awaking a thirst for knowledge.

